# BK virus pneumonia following stem cell transplantation against diffuse large B‐cell lymphoma

**DOI:** 10.1002/rcr2.348

**Published:** 2018-06-26

**Authors:** Akihiro Yoshimura, Taisuke Tsuji, Yuka Kawaji, Yoshiko Hirakawa, Hitoji Uchiyama, Noriya Hiraoka

**Affiliations:** ^1^ Department of Respiratory Medicine Japanese Red Cross Kyoto Daiichi Hospital Kyoto Japan; ^2^ Department of Hematology Japanese Red Cross Kyoto Daiichi Hospital Kyoto Japan

**Keywords:** Autologous peripheral blood stem cell transplantation, BK virus DNA load, BK virus pneumonia, bronchoalveolar lavage, diffuse large B‐cell lymphoma

## Abstract

The patient, a 70‐year‐old woman with diffuse large B‐cell lymphoma (DLBCL), developed haemorrhagic cystitis associated with the BK virus (BKV) and adenovirus type 11. Moreover, chest computed tomography showed ground‐glass opacity (GGO) in the bilateral upper lobe, and we performed bronchoalveolar lavage (BAL). The BKV DNA load was elevated not only in blood but also in BAL fluid (BALF), leading to the diagnosis of BKV pneumonia. After administering cidofovir, the respiratory symptoms and GGO abated. Therefore, detection of BKV DNA in BALF is useful for diagnosing BKV pneumonia. The patient with DLBCL developed BKV pneumonia. We performed BAL, and BKV DNA load was elevated on BALF. The detection of BKV DNA in BALF is useful for diagnosing BKV pneumonia.

## Introduction

The BK virus (BKV) often causes nephropathy in immunocompromised patients but rarely causes pneumonia 1, 2. BK virus pneumonia has been reported most often in children or in acquired immunodeficiency syndrome patients. It is difficult to diagnose BKV pneumonia, which is a rare, fatal disease. Here, we report the case of a patient who developed BKV pneumonia after autologous peripheral blood stem cell transplantation (auto‐PBSCT) against relapsed diffuse large B‐cell lymphoma (DLBCL). The patient was diagnosed with BKV pneumonia by bronchoalveolar lavage (BAL), and the symptoms improved after administration of cidofovir. Therefore, performing BAL proved useful for diagnosing BKV pneumonia.

## Case Report

A 70‐year‐old woman was diagnosed with DLBCL (Ann Arbor Stage: IIIA) and received chemotherapy but relapsed. She was admitted to our hospital in 2017 to undergo auto‐PBSCT after salvage chemotherapy. She suddenly developed grade 3 haematuria on the day of transplantation. We detected a BKV DNA load of 5.0 × 10^7^ copies/mL and adenovirus (ADV) type 11 DNA load of 5.0 × 10^7^ copies/mL in the urine and diagnosed her with haemorrhagic cystitis (HC) associated with BKV and ADV. Although she received immunoglobulin and adenine arabinoside, the HC symptoms did not improve. Moreover, we detected a BKV DNA load of 2.2 × 10^2^ copies/mL in the blood and diagnosed the patient with BK viraemia with complications. Although we administered cidofovir (1 mg/kg, three times a week) from days 8 to 26 post‐auto‐PBSCT, the HC symptoms persisted. The ADV DNA load in urine became negative, but the BKV DNA load in urine did not decrease. Overall, the BKV infection did not stabilize adequately.

**Figure 1:**
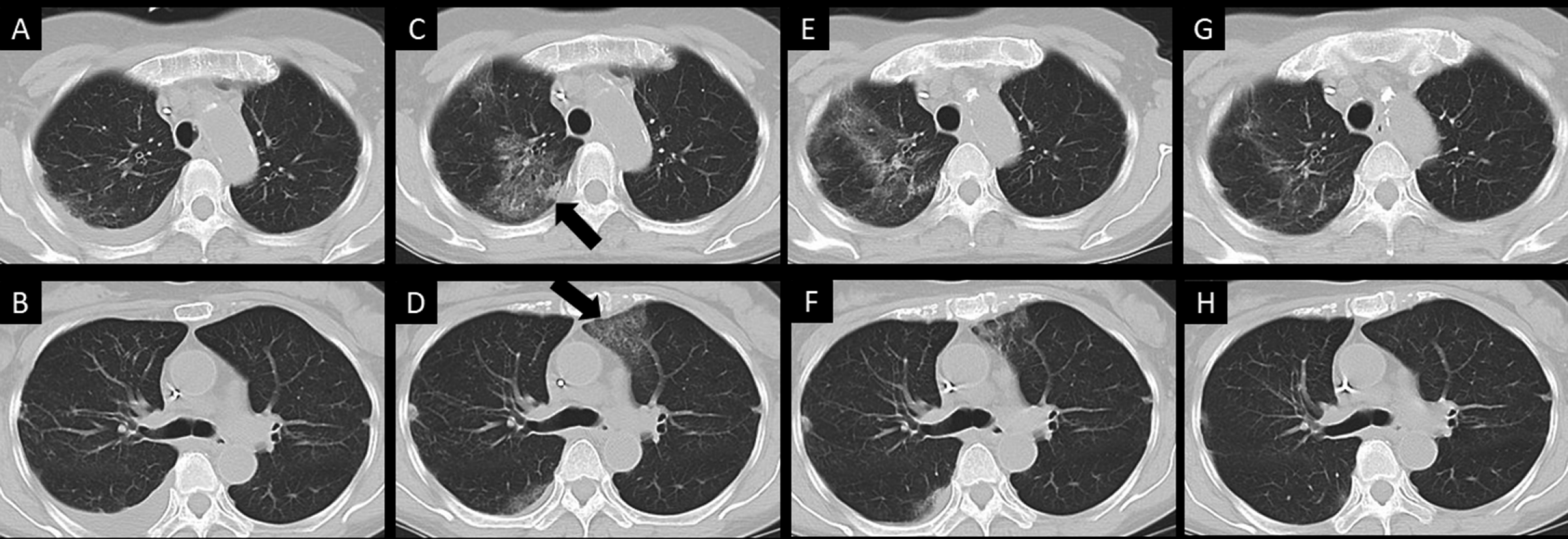
Chest computed tomography (CT). Ground‐glass opacities (GGO) were not observed in panels A and B at day 26 after autologous peripheral blood stem cell transplantation. After nine days, when the patient exhibited respiratory symptoms, GGO was detected in the bilateral lung field (panels C and D). In the CT performed on the seventh day after starting the treatment, the GGO abated. Furthermore, the GGO completely disappeared in the CT performed on the 18th day after starting the treatment.

She exhibited respiratory failure and elevated serum C‐reactive protein levels at day 32 (Table 1). Chest computed tomography (CT) showed ground‐glass opacity (GGO) in the bilateral upper lobe, and we performed BAL at day 34. Although BAL fluid (BALF) was not macroscopically reddish, BAL slightly detected red blood cells on cytology. In BALF, the BKV DNA load was 1.5 × 10^2^ copies/mL, although the ADV and cytomegalovirus DNA loads were not elevated. Although we could not perform lung biopsy because the blood platelet count was low, we diagnosed the patient with BKV pneumonia. After re‐administering cidofovir, respiratory symptoms and GGO in CT abated, although HC symptoms persisted (Fig. 1). The patient has not experienced a relapse of BKV pneumonia and DLBCL even after 11 months.

**Table 1:** Laboratory Data of the Patient at the Onset of Pneumonia

Biochemistry		Serology		Urinalysis	
					
TP	5.8 g/dL	KL‐6	249 IU/mL	SG	1.007
Alb	3.1 g/dL	SP‐A	84.6 ng/mL	pH	7.0
AST	31 IU/L	SP‐D	58.6 ng/mL	Uric blood	(+++)
ALT	13 IU/L	BNP	70 pg/mL	Uric protein	(+)
LDH	224 IU/L	β‐D Glucan	10 pg/mL	Uric sugar	(−)
T‐Bil	0.3 mg/dL	IgG	402 mg/dL	Bil	(−)
BUN	24 mg/dL	IgA	32 mg/dL	RBC	>100/HPF
Cre	1.04 mg/dL	IgM	15 mg/dL	WBC	1–4/HPF
UA	5.1 mg/dL	Procalcitonin	0.17 ng/mL	PCR in urine	
Na	136 mEq/L	MPO‐ANCA	<1.0 EU	BKV DNA	>5.0 × 10^5^ copies/mL
Cl	100 mEq/L	PR3‐ANCA	<1.0 EU	ADV DNA	2.2 × 10^5^ copies/mL
K	4.5 mEq/L	siL‐2R	2270 IU/mL	BALF (Lt, B3b)	
CRP	13.42 mg/dL	Infection		Recovery rate	70/150 mL
Haematology		Anti MAC Ab	(−)	Total cell count	3.4 × 10^5^/mL
WBC	6170 /μL	C7HRP	(−)	Macrophage	76%
Neut.	58.3%	Aspergillus Ag	0.1	Neut.	8%
Lymp.	21.4%	Candida Ag	<0.02	Lymp.	16%
Mono.	19.0%	Cryptococcosis Ag	(−)	CD4/8 ratio	1.08%
Eos.	1.1%	PCR in blood		PCR in BALF	
RBC	259 × 10^5^/μl	BKV DNA	2.4 × 10^4^ copies/mL	BKV DNA	1.5 × 10^2^ copies/mL
Hb	8.3 g/dL	ADV DNA	<1.0 × 10^2^ copies/mL	ADV DNA	(−)
Hct	25.6%			CMV DNA	(−)
MCV	98.8 fl			*Pneumocystis jirovecii* DNA	(−)
MCH	32 pg				
Plt	6.0 × 10^4^/μl				

## Discussion

In this study, the patient underwent auto‐PBSCT after salvage chemotherapy against DLBCL. She developed pneumonia during treatment of BKV infection and was diagnosed with BKV pneumonia following detection of BKV DNA in BALF. Therefore, the detection of BKV DNA in BALF is useful for diagnosing BKV pneumonia.

The BKV, JC virus, and simian virus 40 belong to the non‐enveloped polyoma DNA viruses of the human polyomavirus family 1. The BKV mostly infects the respiratory route during childhood and remains latent in the renal tubule and urothelial cells. In immunocompromised individuals, such as patients with acquired immunodeficiency syndrome and transplant recipients who are undergoing immunosuppressive therapy, BKV reactivates and most often causes nephropathy; BKV nephropathy occurs in up to 8% of the adult recipients of kidney allografts and causes renal dysfunction 1. Due to reactivated BKV spreading through the blood, in addition to nephropathy, BKV causes ureteric stenosis, HC, and pneumonia in a small number of cases 2. Although there is no standard therapeutic agent against BKV infection, cidofovir is one of the effective drugs.

One of the forms of BKV infection, BKV pneumonia is a rare fatal disease that is difficult to diagnose. It is necessary to perform biopsy for a definitive diagnosis of BKV infection. However, thrombocytopenia induced by chemotherapy makes the diagnosis of BKV infection difficult. There was no patient who exhibited improvement of symptoms in the previous six case reports 3. This is the first case, however, where BKV pneumonia was diagnosed during life and improved by cidofovir administration.

The detection of BKV DNA in the urine and plasma is a useful non‐invasive approach for the diagnosis of BKV infection 1. In both immunocompetent and immunocompromised adult patients, BKV is primarily detected in the blood and urine and is not at all detected in BALF 4, and in a previous report, BKV pneumonia was diagnosed based on the positive BKV DNA‐polymerase chain reaction (PCR) results for BALF 3. Therefore, the detection of BKV DNA in BALF should be useful for diagnosing BKV pneumonia.

In the present case, the patient exhibited pneumonia with elevated plasma BKV DNA load after receiving chemotherapy against DLBCL. We did not perform lung biopsy, and the BKV DNA‐PCR results might have been positive due to contamination of blood. However, we detected an elevated BKV DNA load in BALF and the patient’s respiratory symptoms, and the GGO observed in CT abated after re‐administering cidofovir. Therefore, we diagnosed the patient with BKV pneumonia.

It is unclear as to why the symptoms of BKV pneumonia improved, although the HC symptoms did not improve after re‐administrating cidofovir. The BKV DNA load in urine was much higher than in BALF. The response to cidofovir might be different between HC and pneumonia because the cidofovir treatment period for BKV infection has been reported to be significantly associated with BKV DNA load 5.

Although BKV pneumonia is fatal, it is possible to improve the symptoms if the patients are treated early, as in the present case. It should be noted that we performed BAL and detected BKV DNA in BALF as soon as respiratory failure and elevated plasma BKV DNA load were detected. In conclusion, in this study, the patient underwent auto‐PBSCT after salvage chemotherapy against DLBCL. She developed pneumonia during treatment of BKV infection and was diagnosed with BKV pneumonia following detection of BKV DNA in BALF. Therefore, the detection of BKV DNA in BALF is useful for diagnosing BKV pneumonia.

### Disclosure Statement

Appropriate written informed consent was obtained for publication of this case report and accompanying images.
